# A comparative analysis of vitrification and two slow freezing methods for gonocyte-containing neonatal calf testicular tissue and subsequent in vitro culture

**DOI:** 10.1007/s44164-025-00085-8

**Published:** 2025-02-20

**Authors:** Shiyan Tang, Celine Jones, Jill Davies, Sheila Lane, Kevin Coward

**Affiliations:** 1 Nuffield Department of Women’s and Reproductive Health, University of Oxford, Women’s Centre, John Radcliffe Hospital, Oxford, OX3 9DU UK; 2 Wyss Institute for Biologically Inspired Engineering, Harvard University, Boston, MA 02215 USA; 3 Oxford Cell & Tissue Biobank, Children’s Hospital Oxford, Oxford University Hospitals NHS Foundation Trust, Oxford, OX3 9DU UK; 4 Department of Paediatric Oncology and Haematology, Children’s Hospital Oxford, Oxford University Hospitals NHS Foundation Trust, Oxford, OX3 9DU UK

**Keywords:** Immature testicular tissue, Fertility preservation, Vitrification, Slow freezing, Spermatogonial stem cells, Germ cells, In vitro culture

## Abstract

The cryopreservation of neonatal testicular tissue containing gonocytes is crucial for preserving genetic diversity, advancing research, and developing reproductive technologies. In this study, we investigated three cryopreservation techniques, slow freezing (in which the rate of freezing was controlled or uncontrolled) and vitrification, using neonatal bovine testicular tissues containing gonocytes, followed by in vitro culture to evaluate cell functionality. Vitrification resulted in a significantly lower proportion (19.15 ± 1.82%) of seminiferous tubules with > 70% attachment to the basement membrane in comparison to both the controlled slow freezing group (47.89 ± 10.98%) and the uncontrolled slow freezing group (39.05 ± 4.15%) (*P* < 0.05). No significant differences were observed in the proportion of seminiferous tubules containing PGP9.5-positive germ cells when compared between the three methods. Comparable densities of germ cells per unit area were observed in the controlled/uncontrolled slow freezing groups and the vitrification group (7.89 ± 1.83, 7.75 ± 1.75, and 7.92 ± 1.23/104 µm^2^, respectively). In addition, the proportions of Sertoli cells (vimentin-positive) and proliferating cells (Ki67-positive) were similar across the three cryopreservation methods. There were no significant differences in cell membrane integrity and the expression of selected genes when compared between the three cryopreservation groups. Compared to fresh tissue, the uncontrolled slow freezing groups exhibited significantly higher levels of apoptosis (*P* < 0.05); there was no significant change in the controlled slow freezing and vitrification group. Notably, all in vitro cultures of testicular cells, from both fresh and freeze/thawed tissues, displayed the formation of germ cell colonies. Our data demonstrate that vitrification effectively preserves neonatal bovine testicular tissues containing gonocytes, safeguarding cell membrane integrity, promoting proliferation, and protecting against apoptosis. Collectively, these findings propose vitrification as a promising alternative cryopreservation method for immature testicular tissue (ITT) in clinical applications.

## Introduction

In the early postnatal developmental stage of the mammalian testis, gonocytes, which are located in the center of the seminiferous tubules, are the most prominent type of germ cell and are surrounded by Sertoli cells [1]. In bovine and human testes, at approximately 2 months of age, gonocytes originally formed from primordial germ cells (PGCs) gradually begin to migrate to the basement membrane of the seminiferous tubules and develop into spermatogonial stem cells (SSCs) [2, 3]. This transformation is associated with the capacity of SSCs to self-renew and undergo spermatogenesis; consequently, this process is important for the development of male germ cells. Therefore, the preservation of gonocytes during the neonatal period is fundamental for the maintenance of healthy prepubertal testes.

In human and domestic animals, conventional “sperm banking” is not practical for the preservation of fertility during the prepubertal period as the production of functional sperm begins in the peri-puberty period. Therefore, the cryopreservation of immature testicular tissues (ITTs) provides a promising approach to preserve fertility [4, 5]. Gonocytes or SSCs from these cryopreserved immature testicular tissues could be potentially used for future in vitro spermatogenesis to generate sperm to create offspring via assisted reproductive technology (ART).

At present, the most commonly used in vitro cryopreservation methods for the preservation of ITTs involve slow freezing, a technique in which the rate of freezing can either be controlled or uncontrolled. The rate of cooling during slow freezing is crucial because it can affect the rate of formation and the size of ice crystals [6]. Speed-controlled slow freezing allows the application of optimal cooling rates for testicular tissues during cryopreservation and allows us to monitor the actual cooling rate within samples; this method has been used in clinics to preserve human prepubertal testicular tissues. However, speed-controlled slow freezing demands complex and costly equipment; these systems are usually only available in large urban hospitals or central tissue banks. In contrast, uncontrolled slow freezing (e.g., the Mr. Frosty system) represents a cheaper and simpler method, although it is not possible to accurately adjust or design cooling rates that are optimized for different types of tissues.

In our previous study, we recommended that the optimal holding time between tissue collection and cryopreservation should not exceed 24 h after tissue procurement [7]. However, delays in tissue processing are sometimes unavoidable, particularly when tissues are transported over long distances. In some cases, these delays can extend up to 48 h. Consequently, there is a need to implement alternative approaches to cryopreserve ITTs in local tissue banks or fertility clinics prior to transportation in order to reduce tissue holding time and enhance the quality of cryopreserved samples. Vitrification has been proposed as a fast, safe, and efficient fertility preservation technology for use in fertility laboratories for the cryopreservation of oocytes, embryos [8], blastocyst [9], sperm [10], and ovarian tissues [11] with full developmental competence. Vitrification is a physical process in which the liquid in cells and tissues becomes a solid liquid (also referred to as a glass-like state) without the formation of ice crystals; this occurs because the tissue is cooled rapidly [12]. However, vitrification has not been widely used for the storage of ITTs. The use of vitrification to cryopreserve ITTs may enable more timely processing of tissues without the need for a specific machine. Previous research has indicated that vitrification is effective for the cryopreservation of undifferentiated spermatogonia in mice [13], horses [14], and humans [15] in terms of preserving tubule morphology. Vitrified testicular tissues from neonatal boars have been used to produce functional sperm after grafting into nude mice [16]. Furthermore, the completion of spermatogenesis has been reported for both controlled rate freeze/thawed and vitrified neonatal mouse testicular tissues [17]. These studies indicate the significant potential of vitrification for preserving male fertility. However, only a few studies have addressed the cryopreservation of mammalian gonocytes; the vitrification of testicular tissues containing gonocytes has yet to be attempted.

In this study, we compared the efficacy of vitrification, a rapid cryopreservation method, to two traditional slow freezing techniques when used to cryopreserve neonatal bovine ITTs. The bovine model, particularly Holstein bulls, serves as a valuable system for investigating prepubertal testicular development due to its relatively slow testicular maturation from birth to 20 weeks [18] and the initiation of spermatogenesis between 20 and 32 weeks, as well as its structural and microenvironmental similarity to human testes. The first SSCs develop at around 3–4 months after birth in both humans and the bovine model and mainly consist of seminiferous tubules without a central lumen [19]. The three cryopreservation techniques were compared with regard to tubule histology, cell membrane integrity, apoptosis, and gene expression in neonatal gonocyte-containing ITTs. Post-thaw, dissociated cells were cultured in vitro and then analyzed to evaluate the activity and behavior of the testicular cells after cryopreservation.

## Methods

### Collection and preparation of bovine ITTs

Freshly excised testes from 2-week-old Holstein calves (*Bos taurus*) were obtained from Tockenham Corner Abattoir in Swindon, UK. The testes were immersed in basic medium and immediately transferred on ice to our laboratory, where they were processed as previously described [20]. The basic medium was prepared using sterile Hanks’ balanced salt solution (HBSS; Sigma-Aldrich, Missouri, USA) supplemented with 2% penicillin–streptomycin (Sigma-Aldrich), 0.1 M sucrose (Sigma-Aldrich), and 10 mg/ml bovine serum albumin (BSA; Thermo Fisher Scientific, Massachusetts, USA). Isolated ITTs were dissected into fragments approximately 3 × 3 × 3 mm^3^ in size. These fragments were then stratified into three experimental groups based on the cryopreservation technique utilized: controlled slow freezing (in which the rate of freezing was controlled), uncontrolled slow freezing (in which the rate of freezing was uncontrolled), and vitrification.

### Cryopreservation and thawing

For both controlled and uncontrolled slow freezing approaches, a single piece of testicular tissue fragment was submerged in a cryovial containing 1 ml of cryoprotectant medium composed of basic medium and 10% DMSO (Millipore Sigma). In the controlled slow freezing group, cryovials were placed in a programmable freezer (Sy-lab IceCube, Purkersdorf, Austria) and cooled according to a standard clinical protocol for prepubertal human testicular tissue cryopreservation, as established by the Oxford Cell and Tissue Bank (OCTB; Oxford, UK), as described previously [7]. When the program was complete, cryovials were stored in liquid nitrogen (LN_2_, − 196 °C) until further use. For the uncontrolled slow freezing group, cryovials were placed in a Mr. Frosty™ Freezing Container (Thermo Fisher Scientific, Waltham, MA, USA) containing isopropyl alcohol at room temperature, which was then transferred to a − 80 °C freezer overnight before being placed into LN_2_. The thawing procedure used following slow freezing was described previously [20]. In brief, slow-frozen vials were transferred from LN_2_ to a 34 °C water bath for 2 min incubation. Each vial was then immediately diluted by 50% with 1 ml of warm basic medium for 3 min incubation. Subsequently, testicular tissue fragments were washed twice with 3 ml of basic medium with gentle agitation at 34 °C for 5 min.

For vitrification, each single tissue fragment was incubated in 200 µl of equilibration solution (Kitazato, Shizuoka, Japan) for 25 min, followed by 15 min incubation in 200 µl vitrification solution (Kitazato, Shizuoka, Japan). Subsequently, the tissue fragments were rapidly plunged into LN_2_, placed in cryovials, and stored in LN_2_ until further use. To thaw the vitrified samples, cryovials were warmed in a 34 °C water bath, followed by a 1 min incubation in 1.5 ml of pre-warmed thawing solution (Kitazato, Shizuoka, Japan), a 3 min incubation in 600 µl of dilution solution (Kitazato, Shizuoka, Japan), and two 5 min incubations in 600 µl of washing solution (Kitazato, Shizuoka, Japan).

### Histology and immunohistochemical analyses

**Fig. 1:**
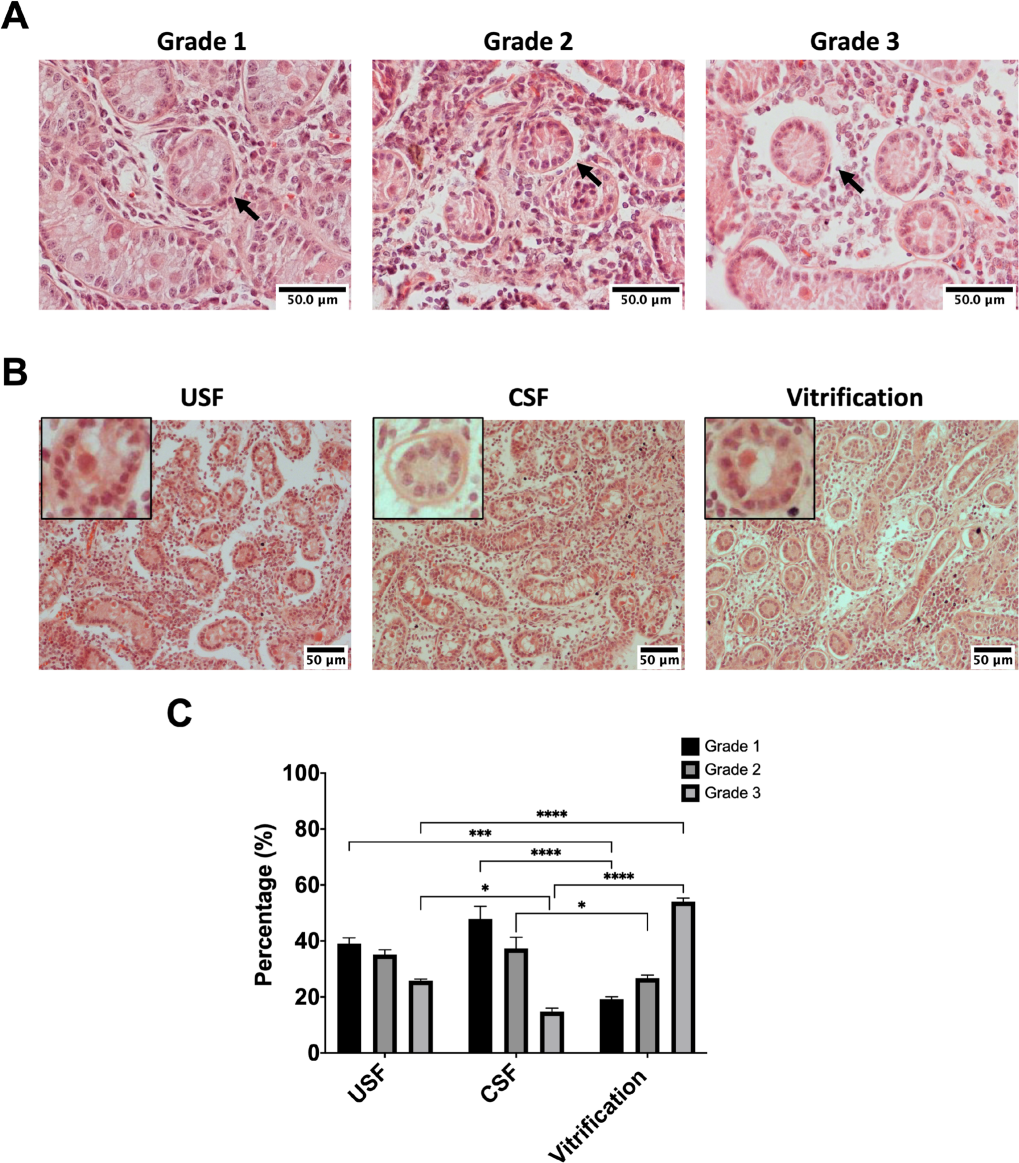
Tissue integrity and histology after vitrification, uncontrolled slow freezing (USF), and controlled slow freezing (CSF). **A** Cross-sectional images of testicular tissue graded 1, 2, or 3 according to adhesion to the basement membrane (BM). Grade 1—“Good”: seminiferous tubules with < 30% detachment of cellular adhesion to the BM. Grade 2—“Satisfactory”: partial detachment (30–70%) detachment from the BM. Grade 3—“Detached”: > 70% detachment from the BM and disrupted cell-to-cell adhesion. **B** Representative hematoxylin and eosin (H&E) images for tissue frozen using the three different cryopreservation methods. The inset in each image displays seminiferous tubules at a higher magnification. **C** Percentage of seminiferous tubules classified as grade 1, 2, or 3. Data were tested for normality using the Shapiro–Wilk test and conformed to a normal distribution. Values represent the mean ± standard deviation (SD) from four technical replicates for each of three biological replicates. G, gonocyte; SCs, Sertoli cells; M, peritubular myoid cell. Scale bar = 50 μm. *P < 0.05, **P < 0.01, ***P < 0.001, and ****P < 0.0001

Freeze/thawed bovine testicular fragments were fixed in 4% formalin (Sigma-Aldrich, St. Louis, USA), embedded in paraffin, and then sectioned into serial Sects. (5 μm thick) using a microtome (Leica, Wetzlar, Germany). Following deparaffinization in xylene and rehydration, tissue slides were stained with hematoxylin and eosin (H&E, MilliporeSigma) to evaluate tubule morphology. The histology of ITTs was categorized as grades 1, 2, or 3 based on their main morphological patterns, structural integrity, and architecture, specifically the attachment of tubule cells to the basement membrane (Fig. 1A), as described previously [7]. Tubules were classified as grade 1 if they exhibited full cellular adhesion or < 30% detachment from the basement membrane, grade 2 if there was partial detachment (30–70% of tubules), and grade 3 if > 70% of tubules were detached from the basement membrane.

For immunohistochemistry, ITT sections were blocked with goat serum for 30 min, followed by overnight incubation at 4 °C with primary antibodies. Anti-protein gene product 9.5 (PGP9.5) (1:100 dilution; ab72911, Abcam, Cambridge, UK) was used to detect germ cells (gonocytes and undifferentiated SSCs), anti-Ki67 (1:100; ab15580, Abcam) was used to detect proliferating cells, while anti-vimentin (1:200; sc-6260, Santa Cruz Biotechnology, Santa Cruz, CA, USA) was used to detect Sertoli cells, as described previously [21]. Sections were then incubated with a secondary antibody (1:200) provided in the VectaStain™ ABC kit (Vector Laboratories, Burlingame, CA, USA), and signals were visualized using 3,3′-diaminobenzidine (DAB; Novus Biologicals, Littleton, CO, USA). All sections were counterstained with hematoxylin. Negative controls were prepared using corresponding concentrations of isotype immunoglobulin-G.

### RNA extraction and quantitative real-time reverse transcription-polymerase chain reaction (qRT-PCR)

Total RNA was isolated from frozen-thawed bovine testicular tissue fragments using the PureLink™ RNA Mini Kit (Thermo Fisher Scientific) in accordance with the manufacturer’s instructions. RNA concentrations were measured using the Qubit® RNA BR Assay Kit (Thermo Fisher Scientific) with fluorescence detection and a Qubit 3.0 fluorometer. RNA integrity was evaluated by gel electrophoresis. The extracted RNA samples were subsequently stored at − 80 °C for future analyses.

Complementary DNA (cDNA) synthesis was performed using the SuperScript™ IV One-Step RT-PCR Kit (Thermo Fisher Scientific, UK), and quantitative real-time PCR (qRT-PCR) was conducted in 96-well plates using the QuantStudio™ 3 system (Applied Biosystems, Foster City, CA, USA), as described previously [7]. In brief, each qRT-PCR reaction comprised a total volume of 20 µl, including 4 µl of diluted cDNA template, 10 µl of Fast SYBR Green Master Mix (Thermo Fisher Scientific), 1 µl of 10 µM forward primer, 1 µl of 10 µM reverse primer, and 4 µl of DNase/RNase-free water. The qRT-PCR cycling conditions were initiated with a pre-denaturation step at 95 °C for 20 s, followed by 40 cycles of amplification (95 °C for 3 s, 60 °C for 30 s). Melting curve analysis was performed at the end of the amplification (95 °C for 15 s, 60 °C for 60 s). All samples were analyzed in triplicate, with a non-template control. β-Actin, validated as a stable reference gene in bovine testis, served as a housekeeping gene. Data analysis was performed using Excel™ (Microsoft, Redmond, WA, USA) and the 2 − ΔΔCt method [22].

Primers are listed in Table 1. To investigate the development of germ cells in neonatal bovine testes, we used a series of germ cell markers (*UCHL-1*, *PLZF*, *GFR*α*1*, *C-KIT*, and *THY1*), pluripotency-associated markers (*OCT-4*, *Nanog*), spermatogenesis markers (*STRA8*, *CREM*, *SOX2*), and a marker of apoptosis (*HSP70-2*).

**Table 1:** Primer sequences used for qRT-PCR in this study. *Stra8*, retinoic acid 8; *Plzf*, promyelocytic leukemia zinc-finger; *Gfrα−1*, glial cell-derived neurotrophic factor (GDNF) receptor alpha-1; *THY1*, thy1 cell surface antigen; *UCHL-1* (also known as *PGP9.5*), ubiquitin C-terminal hydrolase L1; *Oct-4*, octamer-4*; Sox2*, sex determining region Y(SRY)-box g transcription factor 2; *CREM*, cyclic-adenosine monophosphate-responsive element modulator; *HSP* 70–2, heat shock protein *70–2*

Gene	Forward primer (5′–3′)	Reverse primer (3′–5′)
*β-Actin*	TCGCCCGAGTCCACACAG	ACCTCAACCCGCTCCCAAG
*STRA8*	TGTACTCCAGAAACCCCAGTCT	TCCTCTTCTTCTTCCTCCTCAA
*PLZF*	CCAGCAGATTCTGGAGTATGCA	GCATACAGCAGGTCATCCAAGTC
*GFRα−1*	CCACCAGCATGTCCAATGAC	GAGCATCCCATAGCTGTGCTT
*THY1*	TTCATCTCCTTGTGACGGGTT	GCAGAGGTGAGGGAATGGC
*UCHL-1*	ACCCCGAGATGCTGAACAAAG	CCCAATGGTCTGCTTCATGAA
*SOX2*	TTACCTCTTCTTCCCACTCC	TTCTTGCTGTCCTCCATTTC
*OCT-4*	AGAGAAAGCGGACGAGTAT	AGTACAGAGTAGTGAAGTGAGG
*NANOG*	TAAGCACAGGGGGCAAAAGT	ATGGCTAAAAGGGGTGGAGG
*CREM*	TCCGTTATTCAGTCACCACAAA	GAGGGTCTTCGTGAAAGGATTT
*HSP70-2*	TTGGGGACAAGTCAGAGAATG	ATCGTGGTGTTCCTTTTGATG

### Cell viability

After thawing, tissues from the three groups were dissociated to single cells using a two-step enzyme digestion procedure, as described previously [23]. Cell viability was evaluated by trypan blue (Gibco) staining. Equal volumes of cell suspension and trypan blue were mixed; then, the proportion of trypan-blue positive and negative cells was counted using a hemacytometer and a light microscope (Microscope Nikon Eclipse Ti, Japan).

### Assessment of cellular apoptosis

Apoptosis in isolated testicular cells was assessed using terminal deoxynucleotidyl transferase dUTP nick end labeling (TUNEL) staining, following the protocol previously described in our earlier work [20]. In brief, apoptotic cells were detected and quantified by evaluating nuclear DNA fragmentation with a fluorometric TUNEL assay (DeadEnd Fluorometric TUNEL System, Promega, Fitchburg, WI, USA).

Fresh or cryopreserved testicular tissues were digested into single-cell suspensions using a two-step enzymatic digestion method, as described previously [20]. The resulting single-cell suspensions were processed according to the manufacturer’s instructions provided with the TUNEL assay. In brief, cells were fixed with 4% paraformaldehyde in phosphate-buffered saline (PBS) at room temperature, washed, and permeabilized using 0.2% Triton X-100. The TUNEL reaction mixture was prepared and applied to cells, which were then incubated at 37 °C for 60 min in a humidified chamber, protected from light. Fluorescent signals (indicating DNA fragmentation) were visualized and captured using a fluorescence microscope (Nikon, Tokyo, Japan). The number of TUNEL-positive cells was quantified as a percentage of the total cells based on the fluorescence images.

#### In vitro culture and cellular proliferation

Dissociated bovine testicular cells from fresh and frozen/thawed testicular tissues were cultured in 24-well plates at a density of 5 × 10^4^ cells/well at 34 °C in an atmosphere containing 5% CO_2_. The culture medium for each well contained 1 ml of modified Eagle’s alpha medium (MEM-a) supplemented with 10% knockout serum replacement (KSR, Gibco), insulin (10 μg/ml), L-glutamine (2.5 mmol/l), 1 × MEM non-essential amino acids, and penicillin (50 IU/ml). The media in each well was changed every 2–3 days. Germ cells within the colonies were confirmed using two markers (PGP9.5 and OCT4), as described in our previous publication [20]. Images were acquired by microscopy (Microscope Nikon Eclipse Ti, Japan). The number of germ cell colonies was counted, and the diameters of colonies were measured by ImageJ software (National Institutes of Health, Bethesda, MD, USA).

### Data analyses and statistical methods

The normality of raw quantitative data was assessed using the Shapiro–Wilk test. Data that conformed to a normal distribution were analyzed by one-way analysis of variance (ANOVA), followed by Tukey’s post hoc test for multiple comparisons. Data that did not conform to a normal distribution was analyzed by the Kruskal–Wallis test, followed by Dunn’s multiple comparisons test. Statistical significance was set at *P* < 0.05. All statistical analyses were conducted using Prism 9 software (GraphPad, San Diego, CA, USA).

## Results

### Effects of the three cryopreservation methods on histological changes in neonatal bovine ITTs after thawing

The structure of bovine seminiferous tubules in H&E-stained sections was evaluated by light microscopy. A total of 672, 743, and 603 seminiferous tubules were analyzed in the uncontrolled slow freezing, controlled slow freezing, and vitrified groups, respectively. Two-week-old bovine testicular tissues contained interstitial tissue and seminiferous tubules without lumina. Seminiferous tubules consisted of gonocytes located in the center, which were surrounded by Sertoli cells. The integrity of the seminiferous tubules was preserved after freeze/thawing in all groups, with continuous adhesion to Sertoli cells and clear identification between Sertoli cells and germ cells (Fig. 1B). Necrosis was not observed in any of the tissue sections. All seminiferous tubules were categorized into grades 1, 2, and 3 according to adherence between the tubules and the basement membrane. The percentage of grade 1 tubules in vitrified tissues was significantly lower than in the controlled slow freezing or uncontrolled slow freezing tissues (*P* < 0.05), while the proportion of grade 3 tubules was significantly higher in vitrified tissues than in tissues from the uncontrolled slow freezing and controlled slow freezing groups (*P* < 0.05, Fig. 1C). The controlled slow freezing group had the highest proportion of grade 2 tubules.

### Effects of the three cryopreservation methods on germ cells, Sertoli cells, and cellular proliferation

**Fig. 2:**
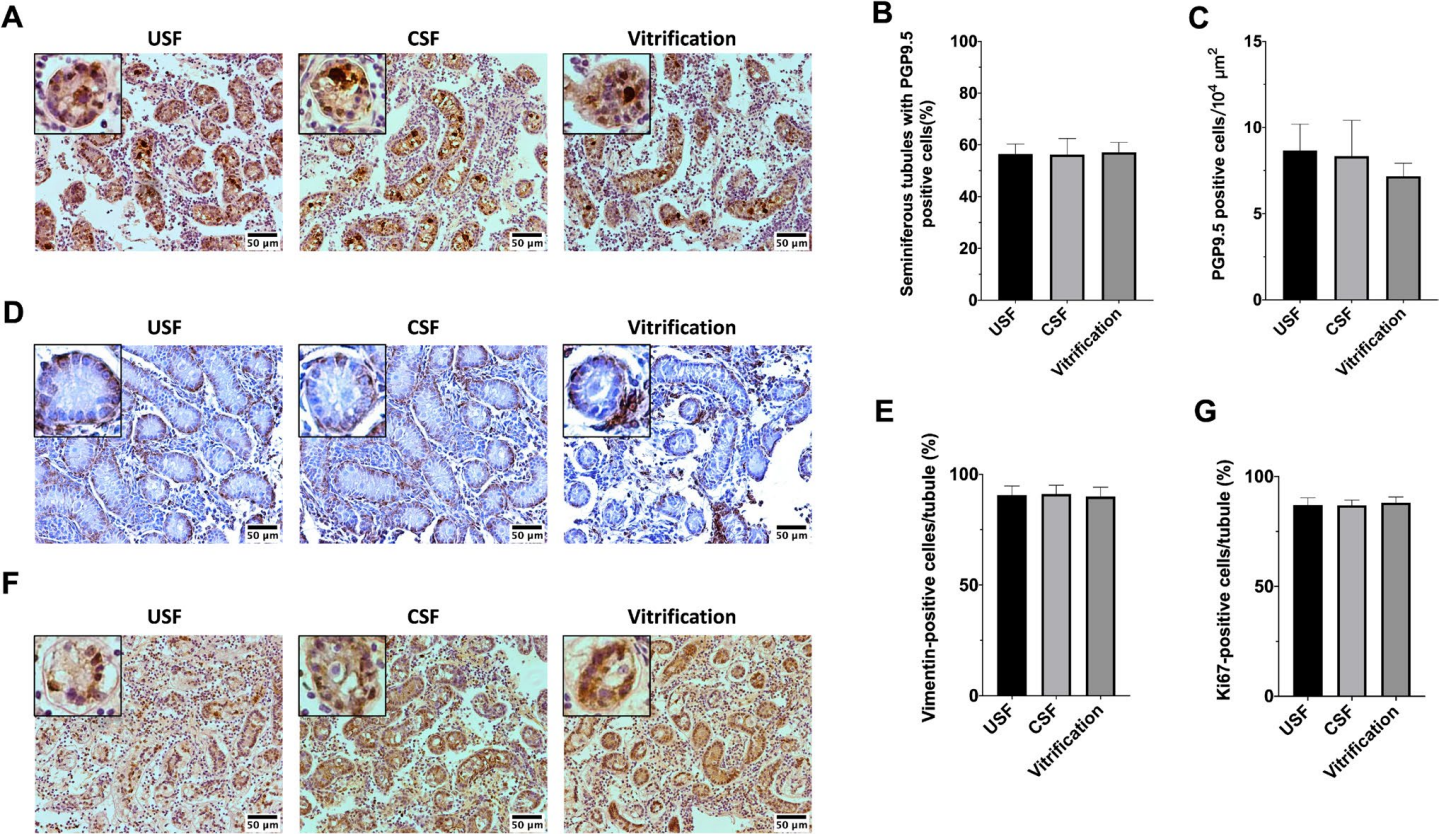
Immunohistochemistry of neonatal testicular tissues after cryopreservation. **A** Representative image of bovine neonatal tissue stained immunohistochemically with the germ-cell marker PGP9.5. **B** The proportion of tubules containing PGP9.5-positive cells. **C** Average number of PGP9.5-positive cells per 10^4^ µm^2^ seminiferous tubules. **D** Representative image of bovine neonatal tissue stained immunohistochemically with the Sertoli-cell marker vimentin. **E** Proportion of vimentin-positive cells per seminiferous tubule. **F** Representative image of bovine neonatal tissue stained immunohistochemically with Ki67, a marker of cell proliferation. **G** Proportion of Ki67-positive cells per seminiferous tubule. The inset in each image displays seminiferous tubules at a higher magnification. Scale bar = 50 μm. USF, uncontrolled slow freezing; CSF, controlled slow freezing. The Shapiro–Wilk test was used to assess normality. For data following a normal distribution, **B** and **E** one-way analysis of variance (ANOVA) was performed with Tukey’s post hoc multiple comparisons; for non-normally distributed data, **C** and **G**, the Kruskal–Wallis test was used, followed by Dunn’s multiple comparisons test. Error bars represent the mean ± standard deviation (SD) of four technical replicates for each of three biological replicates

A total of 495, 520, and 511 seminiferous tubules exhibiting positive staining for the germ-cell marker protein gene product 9.5 (PGP9.5; also known as UCHL-1) were analyzed in the uncontrolled slow freezing, controlled slow freezing, and vitrified groups, respectively. PGP9.5-positive cells exhibited a low cytoplasm-to-nuclear ratio. In two-week-old bovine testes, PGP9.5-positive cells were located in the center of the seminiferous tubules and were in contact with a single layer of surrounding cells (Fig. 2A). Some PGP9.5-positive cells were located between basement cells or located at the basement membrane. The proportion of seminiferous tubules that contained PGP9.5-positive cells per tissue section was 56.44 ± 3.89%, 56.24 ± 6.26%, and 57.10 ± 3.87% in the uncontrolled slow freezing, controlled slow freezing, and vitrified groups, respectively (Fig. 2B). In seminiferous tubules, the number of germ cells per 10^4^ µm^2^ was 7.75 ± 1.75 in the uncontrolled slow freezing group, 7.89 ± 1.83 in the controlled slow freezing group, and 7.92 ± 1.23 in the vitrified group (Fig. 2C): these differences were not significant.

A total of 348, 401, and 394 seminiferous tubules were stained with vimentin (an SC marker) and analyzed in the uncontrolled slow freezing, controlled slow freezing, and vitrified groups, respectively. Vimentin-positive cells were found throughout most of the single cell layer at the basement membrane of seminiferous tubules and in some interstitial cells (Fig. 2D). The proportion of vimentin-positive cells within the seminiferous tubules was 90.61 ± 4.12% in the uncontrolled slow freezing group, 91.11 ± 3.95% in the controlled slow freezing group, and 89.87 ± 4.19% in the vitrified group (Fig. 2E).

A total of 294, 305, and 311 seminiferous tubules stained with Ki67, a marker of cell proliferation, were analyzed in each group. Ki67-positive cells were observed mainly in the nuclei of proliferating Sertoli cells located at the basement membrane of seminiferous tubules and in some interstitial cells (Fig. 2F). Only some germ cells were Ki67-positive. The proportion of seminiferous tubules that contained Ki67-positive cells did not differ significantly when compared between the groups (Fig. 2G). The proportion of Ki67-positive cells within the seminiferous tubules was 87.05 ± 3.30% in the uncontrolled slow freezing group, 86.9 ± 2.40% in the controlled slow freezing group, and 88.12 ± 2.61% in the vitrified group (Fig. 2G).

### Effects of the three cryopreservation methods on changes in the expression of selected genes in bovine ITTs

**Fig. 3:**
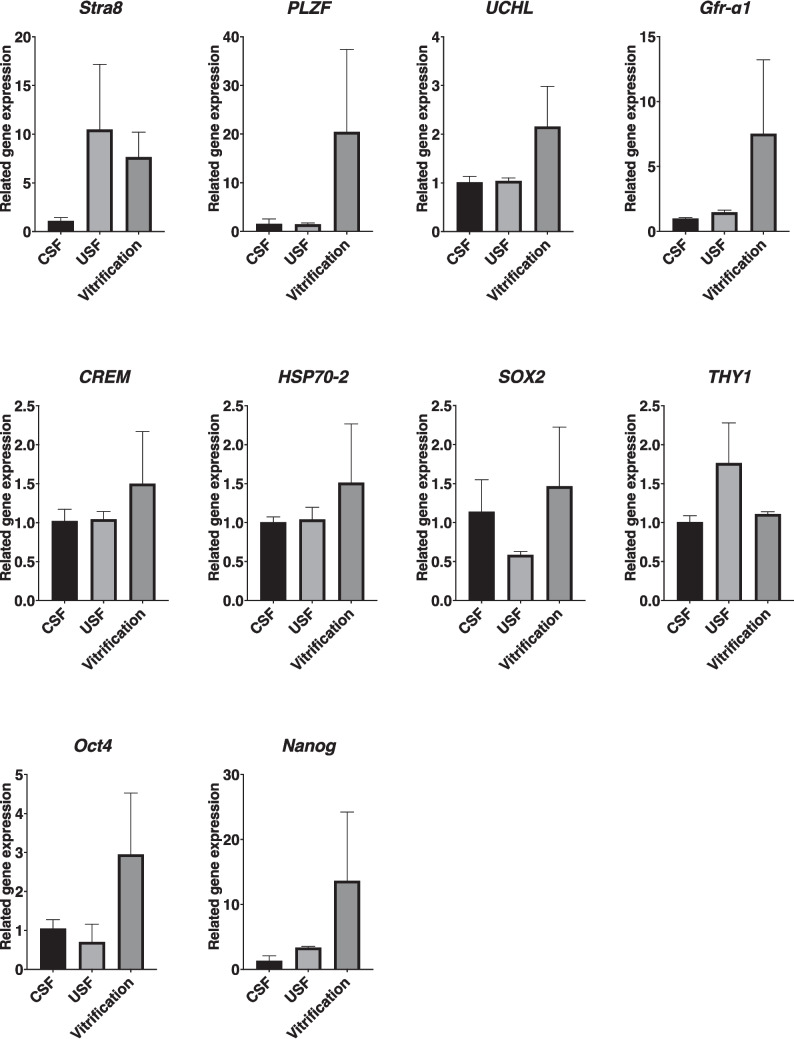
Relative gene expression in immature testicular tissues (ITTs) cryopreserved using three methods. The effects of the three cryopreservation methods, uncontrolled slow freezing (USF), controlled slow freezing (CSF), and vitrification were evaluated by comparing the expression of related genes (*Stra8*, *Plzf*, *UCHL*, *C-kit*, *Gfrα−1*, *CREM*, *HSP70-2*, *Sox2*, *THY1*, *Oct4*, *Nanog*, and *Klf4*) in frozen/thawed bovine ITTs. Data were compared with the Kruskal–Wallis test, and no significant differences in expression between genes were observed. Error bars represent the standard deviation (SD) among biological replicates (*n* = 3)

Next, we used qRT-PCR to detect the expression levels of gonocyte/SSC marker genes (*Gfrα−1*, *Plzf*, *UCHL-1*, and *THY1*), stem-cell markers (*Oct4*, *Nanog*, and *Sox2*), spermatogenesis-related markers (*Stra8* and *CREM*), and the apoptosis-related gene *HSP70-2*, to evaluate the effect of the three cryopreservation methods on ITT quality. No statistically significant differences were detected in the expression levels of any of these genes when compared between the three cryopreservation methods (all *P* > 0.05) (Fig. 3).

### Effects of the three cryopreservation methods on the proportion of membrane-intact live cells and cellular apoptosis

**Fig. 4:**
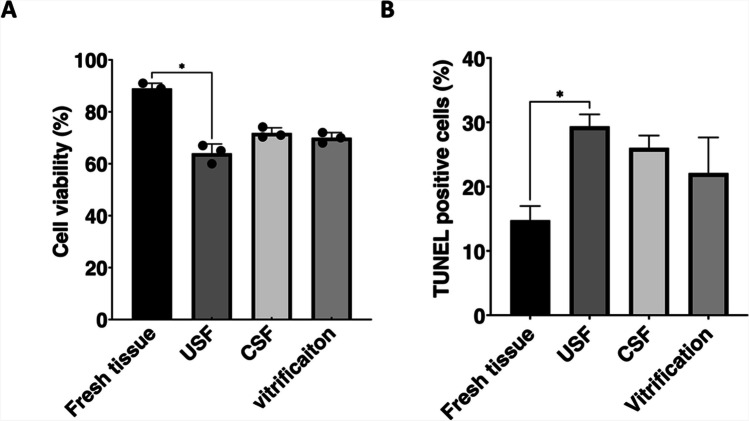
Cell membrane integrity and cell apoptosis in testicular cells from neonatal bovine immature testicular tissue (ITT) samples immediately after collection (fresh) and after cryopreservation by vitrification or uncontrolled/controlled slow freezing (USF/CSF). **A** The proportion of viable cells with intact membrane was evaluated before and after cryopreservation using trypan blue staining. **B** Percentage of TUNEL-positive cells in each of the four groups. Values are the mean ± standard deviation (SD) of three replicates from three donors. *P* < 0.05 was considered significant. Three biological replicates were carried out. *P < 0.05

The membrane integrity of isolated testicular cells (viability) was evaluated immediately after tissue digestion and cell dissociation. The proportion of viable cells in fresh tissue was 89.0 ± 2.0% (Fig. 4A); this was significantly higher than that in the uncontrolled slow freezing group (64.0 ± 3.6%) but not significantly different from the controlled slow freezing (71.8 ± 2.1%) or vitrified groups (70.0 ± 2.0%). There was no significant difference in cell membrane integrity when compared between the three cryopreserved groups.

TUNEL assays were used to evaluate the extent of apoptosis in isolated testicular cells. The percentage of testicular cells that had undergone apoptosis in the uncontrolled slow freezing groups was significantly higher than that in the fresh tissue group (*P* < 0.05) (Fig. 4B); there was no significant difference between the controlled slow freezing, vitrified, and fresh tissue groups (*P* > 0.05).

### Short-term in vitro culture of bovine neonatal testicular cells from frozen/thawed ITTs

**Fig. 5:**
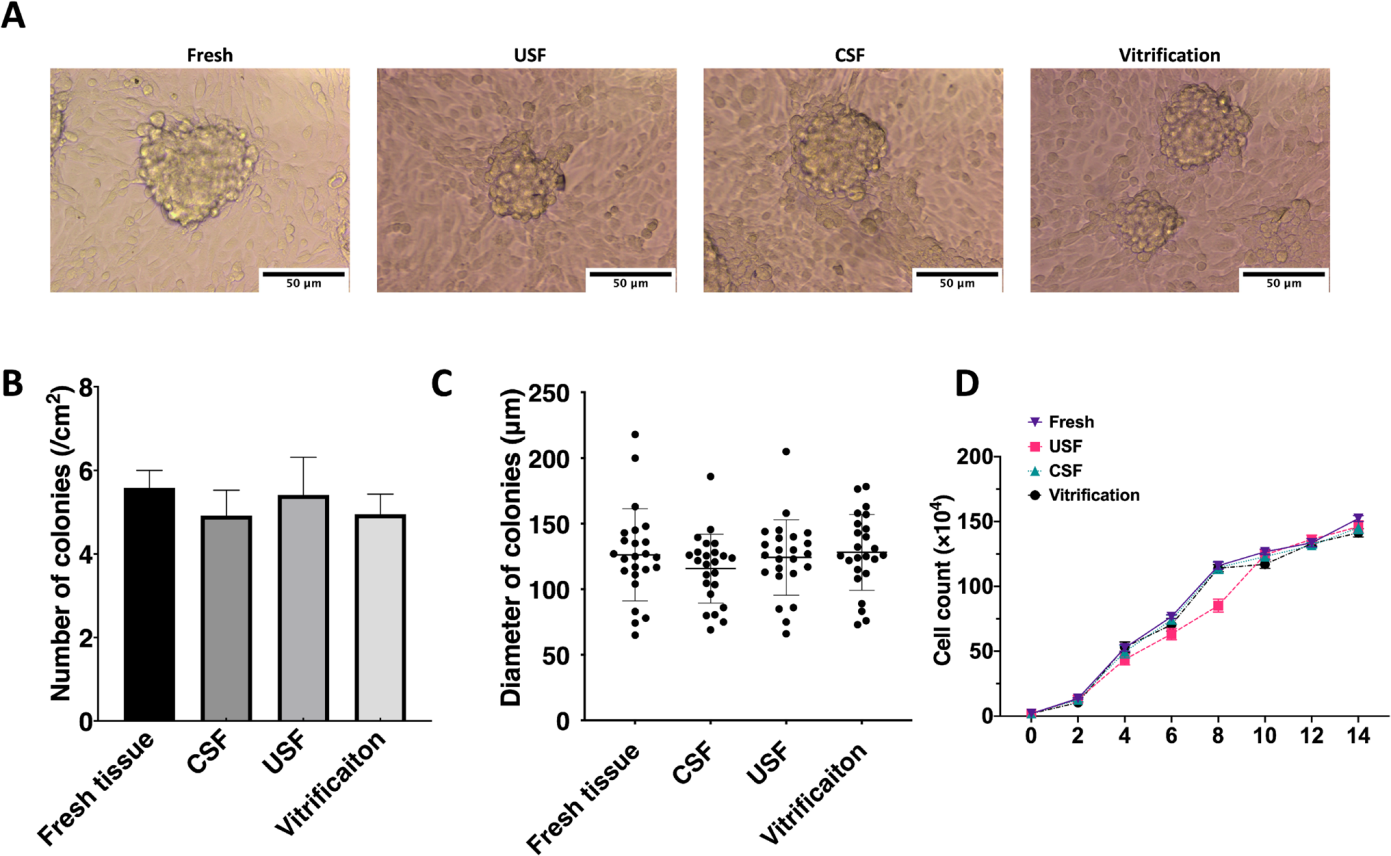
In vitro culture of dissociated testicular cells from tissues cryopreserved using uncontrolled/controlled slow freezing (USF/CSF) and vitrification. **A** Representative bright field images of germ cell colonies at day 7 of in vitro culture; scale bar = 50 μm. **B** Number and **C** diameter of germ cell colonies. **D** Growth curve of in vitro cultured testicular cells from tissues cryopreserved using different methods. Data were tested for normality using the Shapiro–Wilk test and conformed to a normal distribution. Values represent the mean ± standard deviation (SD) from three technical replicates for each of three biological replicates. Statistical analysis was performed using one-way ANOVA with post hoc multiple comparisons

Finally, dissociated testicular cells were cultured in vitro, and cell activity was evaluated. The formation of gonocyte colonies was observed seven days after in vitro culture (Fig. 5A); no differences in the size and number of germ cell colonies were observed. To quantify the effect of the three cryopreservation methods on the formation of germ cell colonies, colony numbers were counted, and the diameter of colonies was measured on day 7. The number of germ cell colonies was 5.58 ± 1.02/cm^2^ in the fresh tissue group, 4.95 ± 1.18/cm^2^ in the vitrified group, 6.91 ± 1.50/cm^2^ in the controlled slow freezing group, and 5.4 ± 2.2/cm^2^ in the uncontrolled slow freezing group 7 days after in vitro culture (Fig. 5B). The morphology and proliferation patterns of germ cell colonies were similar in cells taken from fresh ITTs and frozen/thawed ITTs. The mode of cryopreservation did not significantly affect colony formation in the enriched germ cell cultures. Mean colony diameter was 126.2 ± 35.15 µm in the fresh-tissue group, 115.8 ± 26.22 µm in the controlled slow freezing group, 124.3 ± 28.75 µm in the uncontrolled slow freezing group, and 128.2 ± 28.91 in the vitrified group. A growth curve was drawn for selected cells from ITTs cryopreserved using the three methods: percentage proliferation was similar among the groups and did not differ significantly between groups (Fig. 5D).

## Discussion

The cryopreservation of ITTs may be used to preserve fertility in male patients for whom sperm cryopreservation is not an option. Cryopreserved ITTs can be used for grafting or in vitro spermatogenesis to generate mature sperm at a future date. Therefore, it is crucial to investigate safe and practical methods for the cryopreservation of prepubertal testicular tissue. In this study, we compared three cryopreservation methods (USF, CSF, and vitrification) for the cryopreservation of neonatal bovine testicular tissue. While vitrification led to lower seminiferous tubule attachment to the basement membrane, it preserved germ cells, Sertoli cells, cell membrane integrity, and gene expression in a similar manner to slow freezing. Vitrification also resulted in lower levels of apoptosis when compared to slow freezing and supported the formation of germ cell colonies during in vitro culture. These findings highlight vitrification as a promising alternative for preserving immature testicular tissue in clinical applications.

Controlled slow freezing, uncontrolled slow freezing, and vitrification are the most common methods applied for the cryopreservation of testicular tissues. Slow freezing has been used to preserve ITTs with SSCs in tissue biobanks in both the USA and UK [24, 25]. During controlled slow freezing, the formation of ice within cells is not entirely avoidable and can still cause damage. Vitrification uses a small volume of solution and ultra-rapid liquid solidification, thus avoiding ice formation in cells [26]. Vitrification is also simpler and less expensive than some of the other methods and elicits less damage to cells. Furthermore, portable vitrification devices are available, which make immediate cryopreservation possible for patients in remote areas. This may help to avoid damage to tissue caused by transportation or the inconvenience and potential treatment delays associated with long-distance travel. Therefore, vitrification has been proposed as an alternative method for the cryopreservation of testicular tissues. Live offspring have been produced from vitrified testicular tissues in Japanese quail [27], piglets [28], and neonatal mice [29], highlighting the efficacy and safety of vitrification as a cryopreservation method for ITTs. The production of live offspring from vitrified testicular tissues has been achieved in Japanese quail piglets and neonatal mice, thereby highlighting its efficacy and safety as a cryopreservation method for ITTs. The maintenance of proliferating SSCs from vitrified non-human primate ITTs has been demonstrated in xenografted mice [30]. In addition, vitrified human ITTs (from boys aged 6 and 12 years) exhibited similar spermatogonial and histological tubular structures, thus enabling their survival and proliferation from frozen and fresh samples [15]. Other studies also show similar results from slow freezing and vitrification for cryopreservation of adult and late-prepubertal human testicular tissues [13, 15, 31]. To date, only a limited number of studies have explored the cryopreservation of neonatal testicular tissue containing gonocytes in domestic animals or humans, both of which have a longer prepuberty period and testicular maturation period. Our study addresses the gap in vitrifying the neonatal testicular tissues of domestic animals, specifically bovine tissues containing gonocytes.

The morphology and structure of seminiferous tubules are widely used as indicators of cryopreservation success. In this study, the structural integrity and cellular health of bovine ITTs were evaluated across three cryopreservation techniques using histological analysis. Intact tubule structures with well-defined boundaries were preserved in all cryopreserved tissues, irrespective of the method employed. The tight connections between gonocytes, situated at the center of seminiferous tubules, and Sertoli cells at the basement membrane, were maintained, with no significant gaps observed between these cell types. These findings suggest that both slow freezing and vitrification can effectively preserve the structure of seminiferous tubules. However, an increase in the gaps between seminiferous tubules and interstitial tissue was observed in vitrified tissues, indicating that neonatal seminiferous tubules are more prone to shrinkage when vitrification is applied. This observation aligns with previous findings by Curaba et al., who reported that the primary structural damage in mouse ITTs caused by cryopreservation involved small gaps and an increased number of pyknotic cells within the seminiferous tubules, although detachment from the tubule basement membrane was not specifically investigated in their study [32].

The preservation of germ cells is critical for the successful cryopreservation of immature testicular tissues (ITTs) for reproductive purposes. In this study, we evaluated germ cells, including gonocytes, in seminiferous tubules using PGP9.5 as a marker and found no evidence of germ cell degradation following cryopreservation by controlled slow freezing, uncontrolled slow freezing, or vitrification. Vitrification demonstrated comparable efficacy to traditional slow-freezing methods in the preservation of germ cells within the seminiferous tubules. Sertoli cells, which connect directly to germ cells and provide essential structural support and nutrition, were also well-preserved. Key genes associated with germ cells in Sertoli cells maintained their expression levels, and the structure of thawed tissues remained intact across all cryopreservation methods. Immunohistochemical staining for Ki67 revealed active proliferation in some Sertoli cells and a subset of germ cells, consistent with previous findings in the rat model [33]. Collectively, these results indicate that vitrification, controlled slow freezing, and uncontrolled slow freezing preserve proliferating cells within the seminiferous tubules of neonatal testicular tissues in a similar manner.

We also investigated the expression of gonocyte/spermatogonial stem cell (SSC) markers (*GFRα−1*,* PLZF*, *UCHL-1*, and *THY1*), stem-cell markers (*OCT4*, *Nanog*, and* SOX2*), spermatogenesis-related markers (*STRA8*, *C-kit*, and *CREM*), and the apoptosis-related gene *HSP70-2* following cryopreservation. Collectively, our findings demonstrated that controlled slow freezing, uncontrolled slow freezing, and vitrification provided comparable protective effects on the propagation, self-renewal, pluripotency, differentiation, and stress responses of gonocytes/SSCs in neonatal bovine ITTs. Furthermore, testicular cell activity was assessed using an in vitro culture assay, which evaluated cell membrane integrity, apoptosis, proliferation, and development. Cryopreservation resulted in a reduction in cell membrane integrity and an increase in apoptosis across all techniques; these findings are consistent with those of Milazzo et al. [34] and indicative of tissue degradation during the cryopreservation process. DNA fragmentation, a marker of apoptosis, increased slightly within the normal range after cryopreservation, thus aligning with prior studies on ferret testes [35] and ovarian tissue [36]. Notably, DNA fragmentation has been reported to return to levels similar to fresh tissue after in vitro culture, potentially due to the initiation of DNA repair mechanisms during culture [35]. Despite these challenges, testicular cells from cryopreserved tissues retained the ability to form germ cell colonies and propagated at rates comparable to fresh tissue, thus highlighting the efficacy of all three cryopreservation techniques in terms of the preservation of germ cells. Collectively, these findings, along with histological analyses, confirm the health and functionality of bovine ITTs following cryopreservation.

There are two main limitations to our study that need to be considered. First, we assessed the cryopreservation of neonatal calf testicular tissue only by measuring key parameters after a short period of in vitro cell culture; we did not assess different cell types after in vitro culture, nor did we evaluate the subsequent fertility status of these tissues. Second, we measured the expression of only a few gonocyte-related genes.

In this study, we demonstrated that vitrification provides comparable preservation effects to slow freezing methods on the cell membrane integrity of testicular tissues. Importantly, vitrification offers the advantage of protecting germ cells from damage by preventing ice crystal formation during cryopreservation. While these findings were obtained using the bovine model, further studies, utilizing human neonatal tissues, are essential to comprehensively evaluate the safety and efficacy of vitrification for the preservation of prepubertal human gonocyte-containing testicular tissues. If validated, vitrified tissue grafting or gonocyte transplantation could represent a viable strategy with which to restore fertility in infertile men. Furthermore, vitrification presents a faster, more cost-effective, and accessible alternative for the cryopreservation of testicular tissue, as this technique is already widely employed for embryo and oocyte preservation in IVF clinics. This approach could minimize risks associated with long-term tissue transportation or patient relocation. Furthermore, vitrification holds potential benefits for adult patients unable to produce sperm, thus enabling an efficient option for the storage of testicular tissues for future therapeutic use.

## Conclusion

In this study, three cryopreservation methods were used to preserve neonatal bovine testicular tissues. We observed higher levels of detachment of the seminiferous tubules to the basement membrane in the vitrification group than in the other two slow freezing groups. However, we observed similar effects of vitrification and controlled slow freezing on the preservation of gonocytes and Sertoli cell structure in the seminiferous tubules of neonatal tissue. In addition, vitrification had a similar impact on cell membrane integrity and apoptosis when compared to controlled slow freezing. Moreover, dissociated bovine testicular cells from vitrified and slow-frozen tissues exhibited similar rates of proliferation. The formation of germ cell colonies during in vitro culture was similar across all three cryopreservation techniques. These findings suggest that vitrification could be used as an alternative method to traditional slow-freezing cryopreservation techniques for the long-term storage of early-stage gonocyte-containing bovine ITTs. Our protocol should now be investigated for clinical applications in humans.

## Data Availability

Data are available on reasonable request.
